# Periodic Arrays of Dopants in Silicon by Ultralow Energy Implantation of Phosphorus Ions through a Block Copolymer Thin Film

**DOI:** 10.1021/acsami.3c03782

**Published:** 2023-06-14

**Authors:** Stefano Kuschlan, Riccardo Chiarcos, Michele Laus, Francesc Pérez-Murano, Jordi Llobet, Marta Fernandez-Regulez, Caroline Bonafos, Michele Perego, Gabriele Seguini, Marco De Michielis, Graziella Tallarida

**Affiliations:** † CNR-IMM, Unit of Agrate Brianza, Via C. Olivetti 2, Agrate Brianza I-20864, Italy; ‡ Università del Piemonte Orientale ‘‘A. Avogadro’’, Viale T. Michel 11, Alessandria I-15121, Italy; § Institute of Microelectronics of Barcelona (IMB-CNM, CSIC), Bellaterra 08193, Spain; ∥ CEMES-CNRS, Université de Toulouse, CNRS, Toulouse 31055, France

**Keywords:** block copolymer, ion implantation, doping, silicon, PS-*b*-PMMA

## Abstract

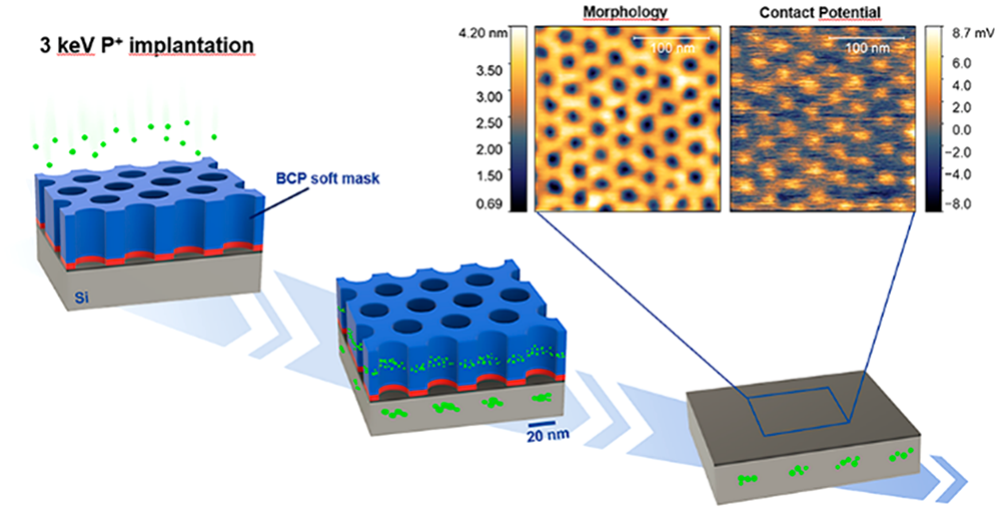

In this work, block copolymer lithography and ultralow energy ion implantation are combined to obtain nanovolumes with high concentrations of phosphorus atoms periodically disposed over a macroscopic area in a p-type silicon substrate. The high dose of implanted dopants grants a local amorphization of the silicon substrate. In this condition, phosphorus is activated by solid phase epitaxial regrowth (SPER) of the implanted region with a relatively low temperature thermal treatment preventing diffusion of phosphorus atoms and preserving their spatial localization. Surface morphology of the sample (AFM, SEM), crystallinity of the silicon substrate (UV Raman), and position of the phosphorus atoms (STEM- EDX, ToF-SIMS) are monitored during the process. Electrostatic potential (KPFM) and the conductivity (C-AFM) maps of the sample surface upon dopant activation are compatible with simulated *I*–*V* characteristics, suggesting the presence of an array of not ideal but working p–n nanojunctions. The proposed approach paves the way for further investigations on the possibility to modulate the dopant distribution within a silicon substrate at the nanoscale by changing the characteristic dimension of the self-assembled BCP film.

## Introduction

Linear block copolymers (BCPs) formed by two different macromolecular chains linked to each other at one end by a covalent bond have been the subject of an intense research activity for a long time, since they provide an attractive and powerful tool for nanoscale fabrication.^1^ Despite their relatively simple structure, when annealed above the glass transition temperature, they spontaneously microphase separate generating a variety of periodic nanostructures, such as spheres, gyroids, lamellae and cylinders. The morphology and characteristic dimensions of the resulting nanostructures can be efficiently tuned by changing the volume fraction (*f*) of the two blocks, the degree of polymerization (*N*), and the Flory–Huggins interaction parameter (χ). The periodicity (*L*_0_) of the microdomains can be varied in the 10–100 nm range by properly adjusting the molecular weight and the interaction parameter of the two blocks.^2,3^ This wide range of possibilities suggested BCPs as fundamental materials for several interesting technological applications. In fact, whenever periodic patterning at the nanoscale over a large surface is required they represent an extremely attractive alternative for lithography^4−7^ and nanotemplating.^8,9^ Possible applications include memories,^10^ sensors,^11,12^ optically active structures,^13,14^ nanoporous membranes,^15,16^ nanocatalysts,^17,18^ and polymer-based photovoltaic cells.^19,20^

The integration of BCP thin films in conventional lithographic processes has been widely explored in the literature^21^ because of the low cost of the self-assembly process if compared to conventional photolithography^22,23^ and the high throughput if compared to serial lithographic processes, such as electron beam lithography (EBL).^24^ After the deposition of the BCP thin film by spin-coating onto the substrate, the self-assembly of the microdomains is usually promoted either via a simple thermal treatment or via a less standard solvent annealing process.^21,25^ The morphology of the nanostructures and their orientation with respect to the underlying substrate are crucial for lithographic applications. In this respect, the most investigated morphologies are the out-of-plane lamellae and cylinders. Accordingly, lamellae or cylinder forming poly(styrene-*b*-methyl methacrylate) (PS-*b*-PMMA) BCPs are considered an excellent and promising candidate for lithographic application since the orientation of the nanodomains with respect to the substrate can be achieved by the robust and simple technique consisting in the formation of a brush layer by means of a random copolymer (RCP) with tailored composition tethered to the sample surface.^26−28^ The BCP film is then spin-coated and self-assembled on top of this neutral brush layer compensating any preferential wetting of the substrate by one of the two blocks forming the BCP. In addition, the registration of the microdomains can be controlled by the so-called “directed self-assembly” (DSA) approach. This process consists in driving the self-assembly of the BCP applying external fields such as electric fields,^29,30^ magnetic fields,^31^ shear forces,^32,33^ or prepatterning the substrate either chemically or topologically.^34,35^ In particular, the prepatterning of the substrate has been widely investigated in BCP lithography: the two approaches are usually referred as chemoepitaxy^36^ when chemical modifications of the surface are used to direct the self-assembly of the BCP or graphoepitaxy^37^ when topological modification of the substrate is exploited.

In order to exploit the self-assembled BCP thin films in a lithographic process, one of the two components of the BCP must be selectively removed from the BCP thin film, generating a nanostructured soft mask on top of the semiconductor substrate. PS-*b*-PMMA BCPs are extremely appealing from this point of view, since the PMMA phase can be selectively removed by means of deep UV exposure and subsequent rinsing in acetic acid, leaving the PS unaffected.^38,39^ Alternatively, the slight difference in etching rates between PS and PMMA components can be exploited to remove the PMMA phase with a dry etching process. Unfortunately, due to low etch resistance of polymeric materials, also the PS film is severely modified during the process, limiting the applicability of this approach.^40^ Similarly, PMMA phase can also be selectively and locally removed using an electron beam to degrade the PMMA and standard development techniques used for EBL resist.^41^ It is worth noting that depending on the specific application, after the selective removal of PMMA, a brief dry etching process may be necessary to remove the RCP neutral layer and to completely expose the underlying substrate. The nanostructured polymer thin film that is obtained upon removal of the PMMA phase can be transferred to the substrate using either subtractive or additive processes. More in detail, the nanostructured polymeric mask can be used for lift-off processes to deposit metals,^42^ oxides, or other materials.^43^ Otherwise, the nanostructured polymer film can be used as a sacrificial layer to pattern the underlying substrate using reactive ion etching.^44^

Surprisingly, to the best of our knowledge, the possibility to use those nanostructured polymeric films as a soft mask to promote local modification of the substrate by conventional ion implantation has not been investigated in detail. In this respect, the poor resistance of polymers to ion bombardment and the limited thickness of these polymer films represent severe limitations and usually relegates those polymer templates to the role of patterning tool for the preparation of hard masks^45−48^ that are subsequently used during the ion implantation process. Alternatively, ultralow energy (*E* < 5 keV) ion implantation represents a viable solution to overcome those limitations. Ultralow energy implantation (1 keV) of Si^+^ ions into a SiO_2_ film through a BCP lithographic mask has already been reported in literature^49,50^ showing that the polymeric film survived the ion implantation process, successfully shielding the substrate. As a result, regular arrays of silicon-rich nanovolumes were formed over the substrate with the same periodicity as the mask and with lateral dimension below 20 nm in the SiO_2_ matrix.

In this work, the combination of ultralow energy implantation of phosphorus ions at high fluences and BCP thin films is investigated to promote a periodic modulation of the concentration of dopant impurities over the near-surface layer of a silicon substrate. The low energy of the implanted ions is expected to preserve the polymer template. Additionally, high implantation doses are considered to induce local amorphization of the silicon substrate. In this way Solid Phase Epitaxial Regrowth (SPER) can be exploited to recover the crystallinity of the silicon matrix by thermal treatments at relatively low temperatures. During the SPER process the phosphorus atoms are substitutionally incorporated into the silicon lattice. This process takes advantage of a low thermal budget to activate the phosphorus atoms preserving their spatial confinement^51^ in a periodic distribution with the nanometric periodicity introduced during the implantation process.

## Experimental Section

### Materials

A −OH terminated poly(styrene-r-methyl methacrylate) P(S-r-MMA) statistical copolymer was prepared by ARGET-ATRP copolymerization (molecular weight *M*_n_ = 3.64 kg/mol, PS fraction *f*_PS_ = 0.61, and polydispersity *Đ* = 1.15).^28^ An asymmetric poly(styrene-*b*-methyl methacrylate) (PS-*b*-PMMA) was bought from “Polymer Source Inc.”. The polymer has *M*_n_ = 67.1 kg/mol, *f*_PS_ = 0.69, and *Đ* = 1.09, resulting in a cylindrical morphology with periodicity *L*_0_ of ∼35 nm.

### Surface Cleaning and Neutralization

A (100) Si wafer doped with boron and nominal resistivity ρ = 0.01–0.05 Ω cm was cleaved in 1 × 1 cm^2^ samples. They were cleaned for 40 min in 80 °C piranha solution (H_2_O_2_ 30% v/v: H_2_SO_4_ 99% v/v, ratio 1:3), rinsed in deionized water (DIW), and dried in N_2_ flux. A 1 wt % solution of the RCP in toluene was spun on the substrates at 3000 rpm for 30 s. The “graft to” reaction was promoted by Rapid Thermal Process (RTP) at 290 °C for 60 s in a N_2_ atmosphere (Jipelec, JetFirst Series rapid thermal processing system).^52^ Ungrafted chains were removed using ultrasonic in toluene for 5 min, samples were dried in N_2_ flux, and the thickness of the resulting brush layer was measured 3.88 ± 0.14 nm with a spectroscopic ellipsometer (J. A. Wollam Co., Inc. M-2000U, xenon lamp, 70° angle of incidence).

### Preparation of the BCP Lithographic Mask

**Figure 1:**
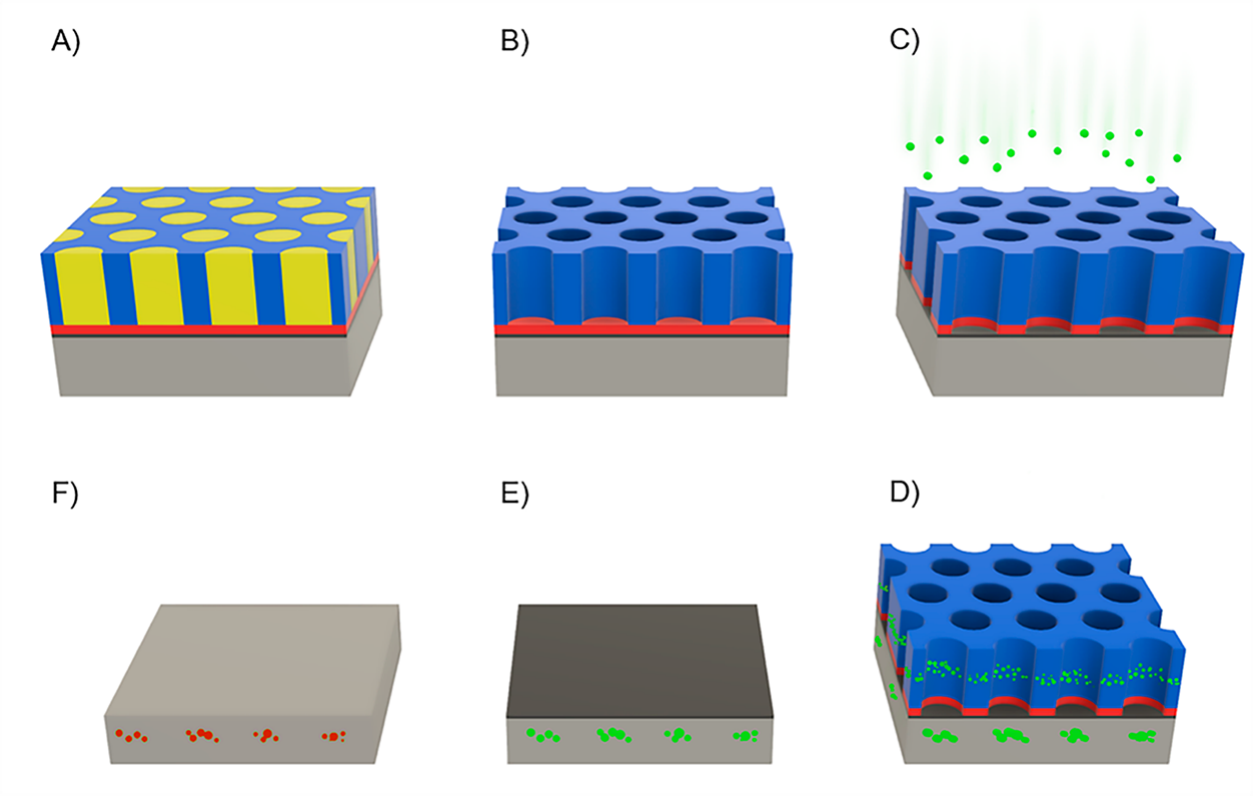
Schematic representation of the process: (A) Self-assembled BCP thin film composed by hexagonally packed PMMA cylinders (yellow) embedded in a PS matrix (blue) and perpendicularly oriented with respect to the substrate. Prior to BCP deposition the not deglazed silicon substrate was neutralized by a random copolymer brush layer (red). (B) Mesoporous polymer film upon selective removal of PMMA phase. C) Removal of RCP brush layer using a mild oxygen plasma treatment, to expose the underlying substrate, and implantation of P^+^ ions (green) at 3 keV. (D) Accumulation of P ions in the PS mask and, in correspondence of the nanopores, in the Si substrate. (E) Removal of the polymeric mask. (F) Removal of the native oxide film by HF treatment and subsequent annealing at low temperature (650 °C) to activate the dopants.

A 1 wt % solution of PS-*b*-PMMA in toluene was spun on top of the neutralized substrates at 2500 rpm for 30 s, resulting in a ∼ 36 nm thick film of BCP. Self-assembly was promoted with an RTP at 230 °C for 300 s in N_2_ atmosphere. The film consists of hexagonally packed out-of-plane PMMA cylinders embedded in a matrix of PS (Figure 1A). Selective removal of the PMMA phase (Figure 1B) was made by exposing the BCP film to UV light (253.7 nm, 5 mW cm^–2^) for 15 min and removing the degraded chains in an acetic acid bath for 8 min. The samples were then rinsed in DIW and dried in N_2_ flux. A mild oxygen plasma treatment (40 W for 96 s) was used to clean the bottom of the pores from the random copolymer RCP brush layer.

### Ion Implantation and Activation of Dopants

Ion implantation was performed on a commercial IMC 210RD medium current ion implanter from Ion Beam Services (IBS). Phosphorus ions were generated from a high purity solid-source material and the dose was calculated from beam current. Ion implantation was performed at 3 keV normal to the samples at different doses of P^+^, ranging from 1.55 × 10^14^ cm^–2^ to 5.02 × 10^14^ cm^–2^ (Figure 1C). Those values were chosen close to the amorphization threshold for P^+^ implantation in silicon, in order to create an amorphous layer at the surface of the samples.^53^ In principle, thanks to the PS mask, P^+^ ions are expected to reach the Si surface only in correspondence of the nanopores, while the rest of the ions are trapped in the polymeric film (Figure 1D). The layer of PS was removed in a piranha solution bath at 80 °C for 40 min, the samples were then rinsed in deionized water (DIW) and dried in N_2_ flux (Figure 1E). The native oxide was removed using a 1% v/v solution of HF for 1 min. Samples were then rinsed in DIW and dried with N_2_. SPER was promoted with a RTP at 650 °C for 10 s in N_2_ atmosphere (Figure 1F).

### Scanning Electron Microscopy

SEM plan view images were acquired using a ZEISS Supra 40 SEM operating at 15 kV. The analysis of the high-resolution plan view images was performed using the software ImageJ. To ensure a statistically relevant analysis, the average diameters of the pores in the mesoporous templates were measured using a set of three images per sample at high magnification (100 000×) and the built-in tool of the software for particles measurement. Similarly, the FFT algorithm of the software was used to measure the periodicity of the nanopores.^52,54^

### Secondary Ion Mass Spectrometry

Time of flight secondary ion mass spectrometry (ToF-SIMS) depth profiling was performed using a dual beam ION-TOF IV system operating in negative polarity. Cs^+^ ions at 1 keV and 110 nA were used to sputter a 300 μm wide square area, while analysis was performed using Ga^+^ ions at 25 keV and 1 pA. Depth scale calibration of phosphorus profiles in the PS matrix was performed using a 50 nm thick PS film spin coated on top of a silicon substrate as a reference to measure the sputtering velocity. Similarly, depth scale calibration in silicon was performed using a 70 nm thick silicon-on-insulator (SOI) sample that was adopted as a reference. Effective concentration of phosphorus in the silicon substrate was performed by calibration of the ^31^P^–^ secondary ion signal using a protocol that is reported in previous publications.^55,56^

### Raman Spectroscopy

UV micro-Raman spectroscopy was used to study the crystallinity of the samples during the process. The set up comprises a Renishaw InVia spectrometer equipped with a frequency tripled Nd:YAG laser (λ = 355 nm), operating with a confocal optical microscope using a 40× objective (NA = 0.47), resulting in a laser spot of ∼1 μm. The power at the sample was measured 4 mW, sufficiently low to avoid the recrystallization of the samples during the measurements. The spectral region between 400 and 600 cm^–1^ Raman shift was considered, because of the characteristic peaks of crystalline silicon (c-Si) at 520 cm^–1^ and the one at 480 cm^–1^ typical of the amorphous silicon (a-Si).^57^ The penetration depth of the laser source is less than 10 nm both in c-Si and a-Si, allowing detection of even a very thin amorphous layer at the surface.

### Transmission Electron Microscopy

High resolution electron microscopy observations and energy dispersive X-ray spectroscopy (STEM-EDS) analyses were performed on a JEOL ARM cold FEG microscope equipped with a probe spherical aberration corrector at 200 kV and a STEM resolution of 78 pm. A CENTURIO-X detector with an elevation angle of 24.3 degrees and a collection angle of 0.98 steradians was used for the EDX measurements. Thin lamellas were prepared for TEM and STEM-EDX analysis using a FEI Helios Nanolab 600i dual beam microscope and focused ion beam (FIB). The cross-sectional lamellas were formed by lowering the voltage from 16 kV-50 pA to 5 kV-15 pA. On the region of interest, a thin (1–2 μm) platinum (Pt) protective layer was deposited. To prevent Pt diffusion, a thin (100 nm) carbon layer was deposited between the sample surface and the platinum protective layer.

### Scanning Probe Microscopy

Several scanning probe microscopy (SPM) setups were used to investigate the surface morphology and the associated electrical properties at the nanoscale. All measurements were carried out in ambient air and at room temperature using the commercial system Bruker Dimension Edge. Atomic force microscopy (AFM) was employed to investigate the surface morphology; measurements were carried out in tapping mode using sharp silicon probes with nominal tip radius of 7 nm and resonance frequency of 300 kHz. Surface electrostatic properties were explored after dopant activation by amplitude-modulated Kelvin probe force microscopy (KPFM); measurements were carried out in lift-mode using PtSi silicon probes by Nanosensors. At each scan line the surface morphology profile was detected first, then the probe was lifted above the surface by tens of nanometers while an AC voltage is applied directly to the probe, making it responsive to the local electrostatic forces. A dedicated KPFM feedback loop was used to generate at each point the potential that must be applied to the probe in order to minimize these forces; this potential, usually named contact potential difference (CPD), provided information on the electrostatic landscape of the sample surface in relation to its morphology.^58^ Finally, local conduction properties were investigated by Conductive-AFM (C-AFM); measurements were taken in contact mode using doped diamond probes (Nanosensors), while a DC voltage bias was applied to the sample. Current was collected either concurrently with surface morphology, thus providing current maps at fixed voltage, or in spectroscopic mode at fixed surface positions while sweeping the applied voltage. Data analysis of SPM measurements was carried out by Gwyddion software.^59^

### Simulations

Simulation results on a model of the sample were obtained by exploitation of the commercial software COMSOL (Multiphysics), where the finite element method (FEM) is used to numerically solve (partial) differential equation systems. This software was set to compute the solution of the Poisson equation coupled to the drift-diffusion equations for both electrons and holes in semiconductors. Boundary conditions were applied to obtain a solution of the equation system and the resulting potentials were then used to estimate the charge current flowing through the device when electrostatic potentials were applied.

## Results and Discussion

**Figure 2:**
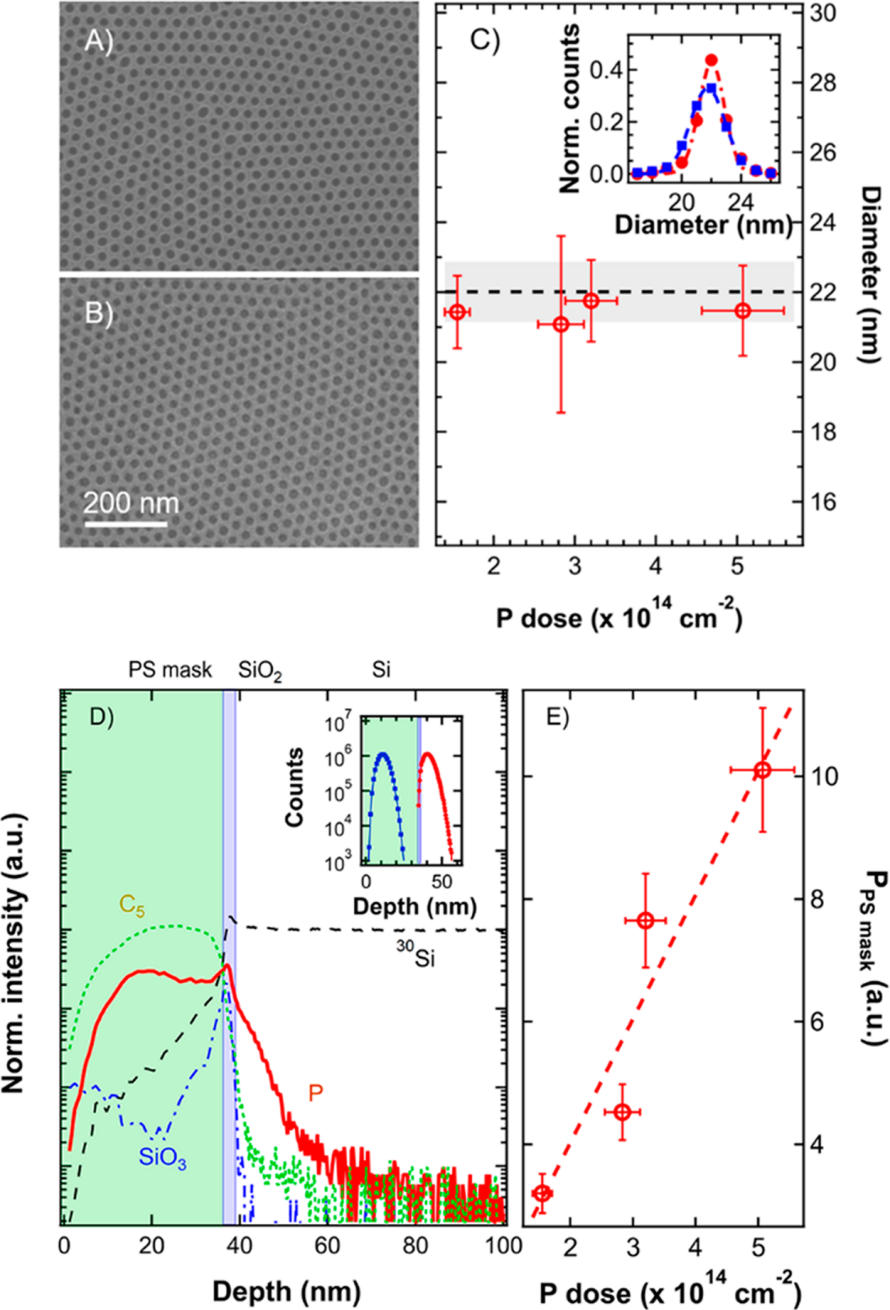
SEM images of the PS mask (A) before and (B) after the implantation, P fluence of 3.20 × 10^14^ cm^–2^. (C) Diameter of the pores as a function of the P dose implanted. The average diameter of the PS mask before implantation is reported for comparison (black dashed line), along with its variation (gray area). In the inset, the distribution of diameters and corresponding Gaussian fit before (red bullets and dashed line) and after (blue squares and dashed line) the implantation is reported for the fluence 3.20 × 10^14^ cm^–2^. (D) Normalized ToF-SIMS depth profile of a sample after the implantation at a dose of 3.20 × 10^14^ cm^–2^. The graph reports the P, C_5_, ^30^Si, and SiO_3_ signals. It is possible to discern between the PS mask, the thin layer of native oxide and the crystalline bulk of silicon (respectively the light green, light blue and white areas). Due to the porosity of the mask, signals in the light green area have both the contribution of the PS and a small contribution from the layers below. A simulation of the implantation profile in nondeglazed Si (red bullets) and in PS (blue squares) is reported in the inset. To better compare the results, the two curves were shifted along the *x*-axis to match the different layers of the ToF-SIMS profile. The simulation was made using the software SRIM13. (E) P dose inside the PS mask as a function of the implanted dose of P.

By grafting the hydroxy terminated poly(styrene-r-methyl methacrylate) P(S-r-MMA) statistical copolymer on the not deglazed Si surface, a neutral brush layer was formed on top of 1 × 1 cm^2^ samples that were cut from a p-type Si (100) wafer. Cylinder forming PS-*b*-PMMA thin films were self-assembled on top of the brush layer, achieving perpendicular orientation of the hexagonally packed PMMA cylinders with respect to the underlying substrate. Upon removal of the PMMA phase, the mesoporous PS templates were implanted with P^+^ ions at 3 keV. The integrity of the mesoporous PS film upon implantation was accurately monitored to confirm its effectiveness in shielding the substrate. Several SEM plan view images of the pristine mask were taken for each sample and compared with those acquired right after the ion implantation. Representative SEM images of the polymeric template before and after ion implantation (3 keV, 3.20 × 10^14^ cm^–2^) are reported in Figure 2A and B, respectively. At a first glance, no significant evidence of ion implantation related damage can be detected on the surface of the mask and the morphology appears unaffected by the implantation. A more accurate analysis of the samples was performed by measuring the diameters of the pores and their size distribution before and after ion implantation for the different implantation conditions. The distribution of pore diameters for each sample was determined by analysis of the collected SEM images, following a protocol that is described in more details in a previous publications.^54,60^ The inset of Figure 2C shows the distributions of pore diameters for the pristine mask and the sample implanted with P ions at 3 keV and dose 3.20 × 10^14^ cm^–2^. The diameter distributions were fitted with Gaussian curves (solid lines) to determine the average diameter of the pores. This analysis was performed on all the implanted samples (Figure S1) and the average diameters are reported as a function of the dose of implanted phosphorus ions in Figure 2C. Accordingly, the pores in the pristine template have an average diameter *D*_0_ ∼ 22 ± 1 nm and a center-to-center distance *L*_0_ ∼ 35 ± 1 nm, consistently with data previously reported in the literature.^60^ Interestingly, data indicate that, within the experimental error, ion implantation did not remarkably change the distribution of the pore diameters. However, after implantation the average values of the pore diameter are systematically lower of almost 1 nm than in the pristine mesoporous template. This slight reduction of the diameter may be due to the swelling of the mesoporous polymer template, because of phosphorus ion incorporation into the PS matrix. The distribution of the pore diameter, expressed by the standard deviation of the Gaussian fit of the experimental data, remains almost constant at each dose, compared to the pristine mask. The only exception is made by the sample implanted at 2.83 × 10^14^ cm^–2^ that shows a different behavior. This deviation can be related to the presence of more defects in the PS mask, or a more inhomogeneous swelling caused by the ions in the polymeric film.

The distribution of phosphorus atoms in the samples after ion implantation was investigated by ToF-SIMS depth profiles. Figure 2D shows the ToF-SIMS depth profile of the samples implanted with P ions at 3 keV and dose 3.20 × 10^14^ cm^–2^. The C_5_^–^, SiO_3_^–^ and ^30^Si^–^ secondary ion signals are reported as markers of the mesoporous PS film, the thin native oxide layer and the silicon bulk, respectively. It is worth noting that, due to the porosity of the polymer film and the large area investigated by ToF-SIMS, the secondary ion signals of the topmost layer of PS are partially overlapped with those of the underlaying SiO_2_ and Si layers. This is particularly evident looking at the SiO_3_^–^ and ^30^Si^–^ secondary ion signals, exhibiting a long tail in the PS mask. Consequently, it is very hard to isolate the P^–^ secondary ion signal originated from the P ions trapped in the mask, preventing the possibility to provide a precise calibration of the P^–^ secondary ion signal in the PS matrix. Nonetheless, the presence of an intense P^–^ secondary ion signal in the PS matrix, with a maximum located ∼15 nm below the surface of the polymer film, suggests that effective P ion accumulation occurred in the mesoporous PS template providing clear indication that the mask was able to retain a certain amount of P ions during the implantation process at low energy. Integrating the phosphorus secondary ion signal in the PS mask region (Figure S2) it is possible to obtain a qualitative estimation of the ions blocked by the polymer as a function of the total dose of phosphorus implanted as shown in Figure 2E. Even if a quantitative analysis of the ToF-SIMS data is prevented in this system, experimental data provide clear evidence of a linear increase of the amount of P ions trapped into the PS matrix when increasing the dose of implanted P ions. This result indicates that, in this range of doses, the mask is effectively shielding the underlying Si substrate.

**Figure 3:**
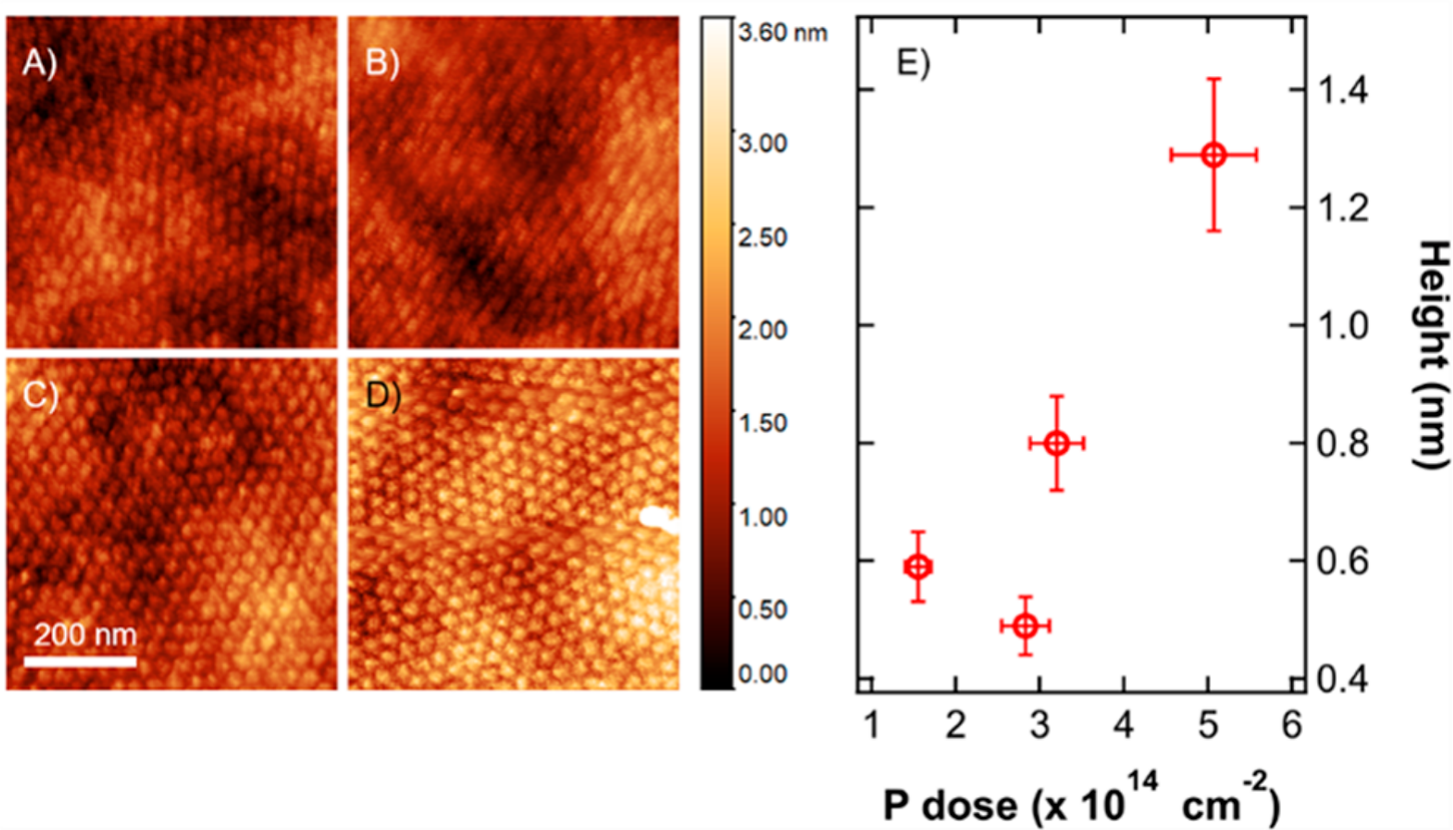
AFM image of the Si surface after the implantation and removal of the mask. Each image corresponds to a different phosphorus dose: (A) 1.55 × 10^14^ cm^–2^, (B) 2.83 × 10^14^ cm^–2^, (C) 3.20 × 10^14^ cm^–2^, and (D) 5.07 × 10^14^ cm^–2^. (E) Average height of the local swelling induced by the ion implantation as a function of the P dose, estimated after the removal of the long-range corrugation of the Si surface.

After the removal of the polymeric mask, the surface morphology of the silicon substrate was investigated by AFM. Figure 3A shows topographic AFM images of the implanted samples. A topographic image of a pristine Si substrate is shown in Figure S3 as a reference. Before implantation the sample exhibits a flat surface with negligible roughness. Conversely, AFM images in Figure 3A–D clearly show that surface morphology of the samples is significantly modified upon implantation, with the appearance of hexagonally packed circular swollen areas, whose organization and periodicity perfectly match the pattern of the mesoporous PS template. This change of the surface morphology further confirms the efficacy of the mesoporous PS template as soft mask to shadow the underlying substrate. Analyzing the images and taking into account the roughness of the sample, it was possible to measure the average heights of the swollen areas that are reported as a function of the implanted dose in Figure 3E. According to the collected data, an increase in the height of the implanted areas is observed, increasing the amount of implanted phosphorus ions.

**Figure 4:**
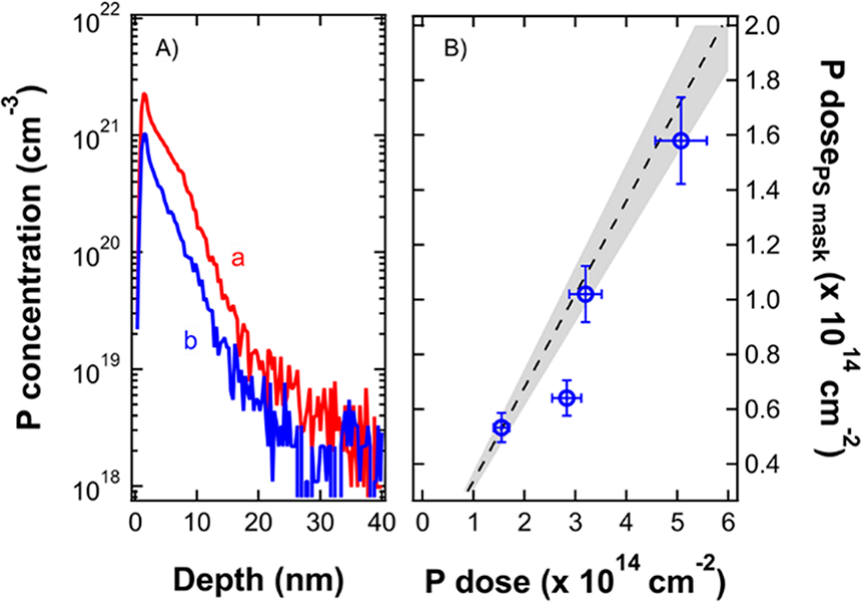
(A) Calibrated ToF-SIMS depth profiles of phosphorus implanted in Si at 3.20 × 10^14^ cm^–2^ dose. Red curve (a) refers to a sample implanted without the PS mask and blue curve (b) with the mask. (B) P dose implanted in Si through the PS mask as a function of the dose of implanted P ions. Black dashed line shows the expected dose with error (gray area) inferred from the analysis of SEM images of the PS mask.

Upon removal of the mesoporous PS template, calibrated depth profiles of the phosphorus atoms implanted into the silicon substrate were acquired by ToF-SIMS analysis for each sample. Figure 4A reports the calibrated phosphorus depth profiles for two samples implanted directly into the silicon substrate (red curve) and without (blue curve) the polymeric mask with the same dose of P ions corresponding to 3.20 × 10^14^ cm^–2^. The different concentration of phosphorus in the two samples is attributed to the shielding of the substrate by the mesoporous PS template. Integrating the calibrated phosphorus depth profiles, the doses of implanted phosphorus ions are determined in the two samples. The ratio between the calculated doses is ∼0.3. This result is perfectly consistent, within the experimental error, with the ratio between the effective surfaces that were exposed during ion implantation in the two samples. Figure 4B reports the phosphorus doses implanted in the samples through the mesoporous PS mask as a function of the doses implanted in samples without the mask. The dashed line indicates the expected dose of phosphorus atoms calculated considering that the exposed area in the masked sample is ∼0.34. The measured doses are in good agreement with the expected ones. This result further confirms that the mesoporous PS template effectively shielded the substrate from the impinging ions, leading to localized implantation of the phosphorus ion in correspondence of the pores in the PS film.

**Figure 5:**
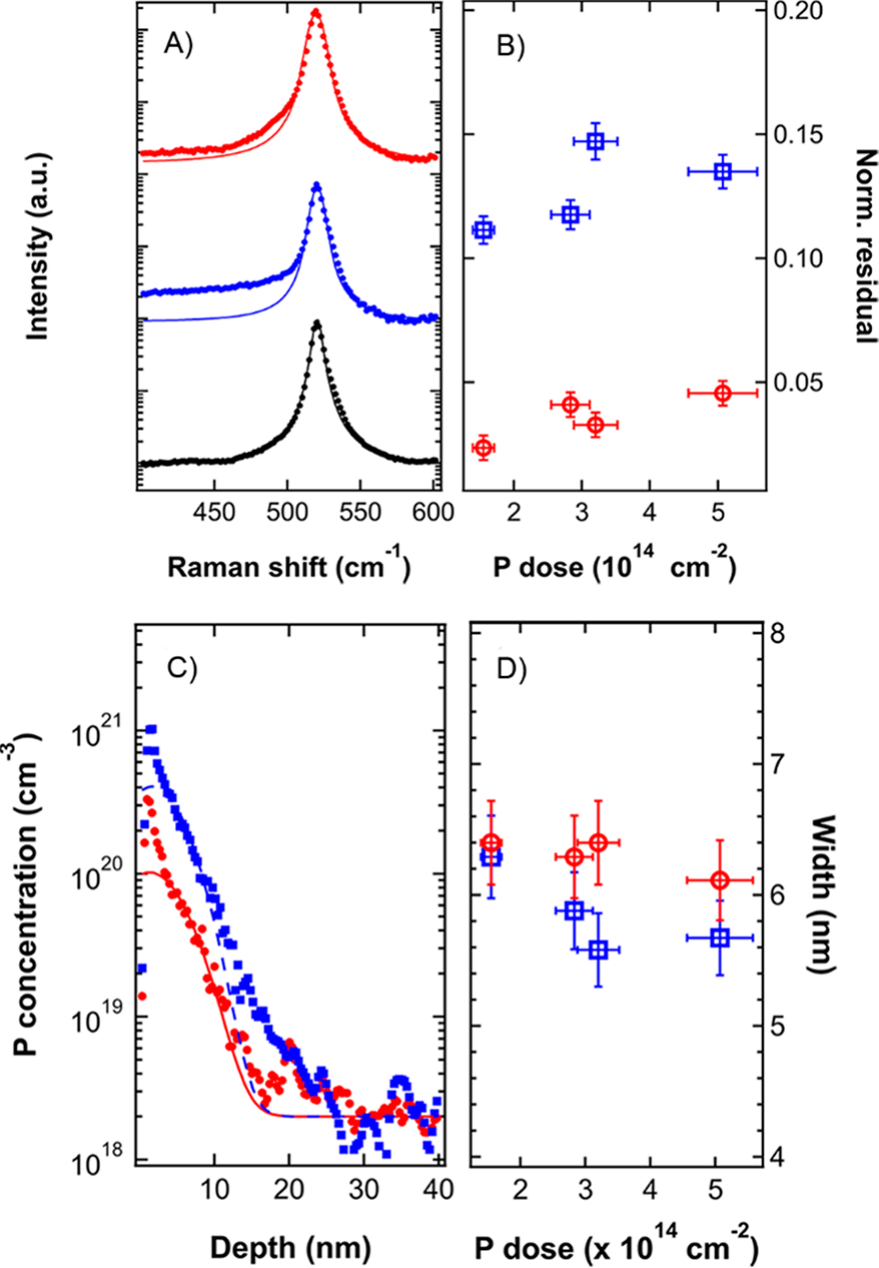
(A) Raman spectra of the sample implanted with a dose of 3.20 × 10^14^ cm^–2^ without the PS mask before implantation (black bullets), after implantation (blue bullets), and upon annealing at 650 °C for 10 s (red bullets). Experimental data are fitted with a Voigt function (solid lines). (B) Normalized residuals in the 400–500 cm^–1^ interval as a function of the implantation fluence before (blue squares) and after (red circles) the annealing. (C) Calibrated ToF-SIMS depth profiles of phosphorus implanted in Si with the PS mask, dose 3.20 × 10^14^ cm^–2^. Blue dots refer to the sample before the annealing, while red squares refer to the sample after the implantation. Experimental data were fitted with a Gaussian curve (blue dashed line before annealing, red solid line after the annealing). (D) Standard deviation of the Gaussian fits as a function of the phosphorus dose before the annealing (blue squares) and after (red circles).

The activation of the dopants upon implantation is commonly achieved by thermal treatments performed in an inert atmosphere at high temperatures. The phosphorus depth profiles for the sample implanted with a phosphorus dose 1.55 × 10^14^ cm^–2^ before and after different high temperature annealing processes are reported in Figure S5. Significant in-depth diffusion of the dopants was observed. Accordingly, the process is expected to promote in-plane diffusion of the dopants in the samples implanted throughout the mesoporous PS template, losing control on the spatial localization of the phosphorus atoms. It is worth noting that in our system the very high phosphorus doses are expected to induce a local amorphization of the silicon substrate. The amorphization of the implanted region was investigated by Raman spectroscopy. Figure 5A shows the Raman spectra before (black symbols) and after (blue symbols) the implantation process for the sample implanted without any polymeric mask with a P dose of 3.20 × 10^14^ cm^–2^. Before implantation, the silicon crystal exhibits a sharp peak centered at 520 cm^–1^ that can be fitted with a single Voigt curve. This peak is typically associated with the *O*(Γ) phonons of the crystalline silicon.^57^ After implantation the Raman spectrum of the sample is characterized by a long tail in the region 400–500 cm^–1^. The presence of amorphous silicon is usually associated with the presence of a broad peak at ∼480 cm^–1^ in the Raman spectrum.^61^ Due to the limited penetration of the P ions in the silicon substrate when operating at 3 keV, the thickness of the amorphized region is anticipated to be lower than 10 nm. The signal generated by this very thin layer is expected to be very broad, making difficult to clearly resolve the two peaks at 520 and 480 cm^–1^. The increased intensity of the signal in the 400–500 cm^–1^ range is associated with the presence of an a-Si layer at the surface. Accordingly, P activation in the implanted region can be achieved by a thermal treatment at relatively low temperature, taking advantage of SPER to recover the crystallinity of the silicon matrix and to concomitantly incorporate the phosphorus atoms in substitutional sites of the silicon crystal promoting their activation. Figure S4 shows representative Raman spectra of an implanted sample after removal of the native oxide layer and subsequent annealing at temperatures ranging from 550 to 1100 °C. No shift of the position and no broadening of the signal associated with crystalline silicon is observed irrespective of the annealing temperatures. The tail in 400–500 cm^–1^ region is progressively reduced as the annealing temperature increases. This reduction is assumed to be indicative of the recrystallization of the amorphous regions. According to these data the threshold temperature to achieve an almost complete recrystallization of the silicon substrate without significant P diffusion is identified to be ∼650 °C. Figure 5A shows the Raman spectrum upon annealing at 650 °C (red symbols) of the sample implanted without any polymeric mask with a P dose of 3.20 × 10^14^ cm^–2^. The symmetry of the peak is almost completely recovered. Fitting the Raman spectra with a Voight function centered at 520 cm^–1^ and with fwhm equivalent to the one obtained in the case of the pristine silicon substrate it is possible to define a parameter that provide a direct indication of the presence of an amorphized region in the silicon substrate by integrating the residuals of the fitting procedure in the 400–500 cm^–1^ region. These values, normalized on the intensity of the Voight function centered at 520 cm^–1^, are reported in Figure 5B for all the implanted samples before (blue symbols) and after (red symbols) annealing at 650 °C. The two sets are characterized by a significant reduction of the calculated values upon annealing, suggesting an almost complete recrystallization of the samples.

To confirm the limited diffusion of phosphorus during the low temperature thermal treatment, calibrated phosphorus depth profiles of each sample upon annealing at 650 °C were acquired by ToF-SIMS analysis and compared with the corresponding calibrated phosphorus depth profiles obtained before the annealing. Figure 5C shows two representative calibrated phosphorus depth profiles before (blue symbols) and after (red symbols) annealing for the sample implanted through the mesoporous PS template with a phosphorus dose of 3.20 × 10^14^ cm^–2^. Due to the removal of the native oxide before the annealing and the subsequent oxidation of the surface, the calibrated phosphorus depth profiles upon annealing is shifted toward the surface along the *x*-axis. The profiles were fitted with a Gaussian curve. The standard deviations of the fitting curves for all the implanted samples before (blue symbols) and after (red symbols) annealing are reported as a function of phosphorus dose in Figure 5D. The standard deviation is almost constant for all the implant doses. Moreover, the slightly large values obtained upon annealing indicate that the 650 °C thermal treatment introduces no significant variation in the phosphorus depth profile.

**Figure 6:**
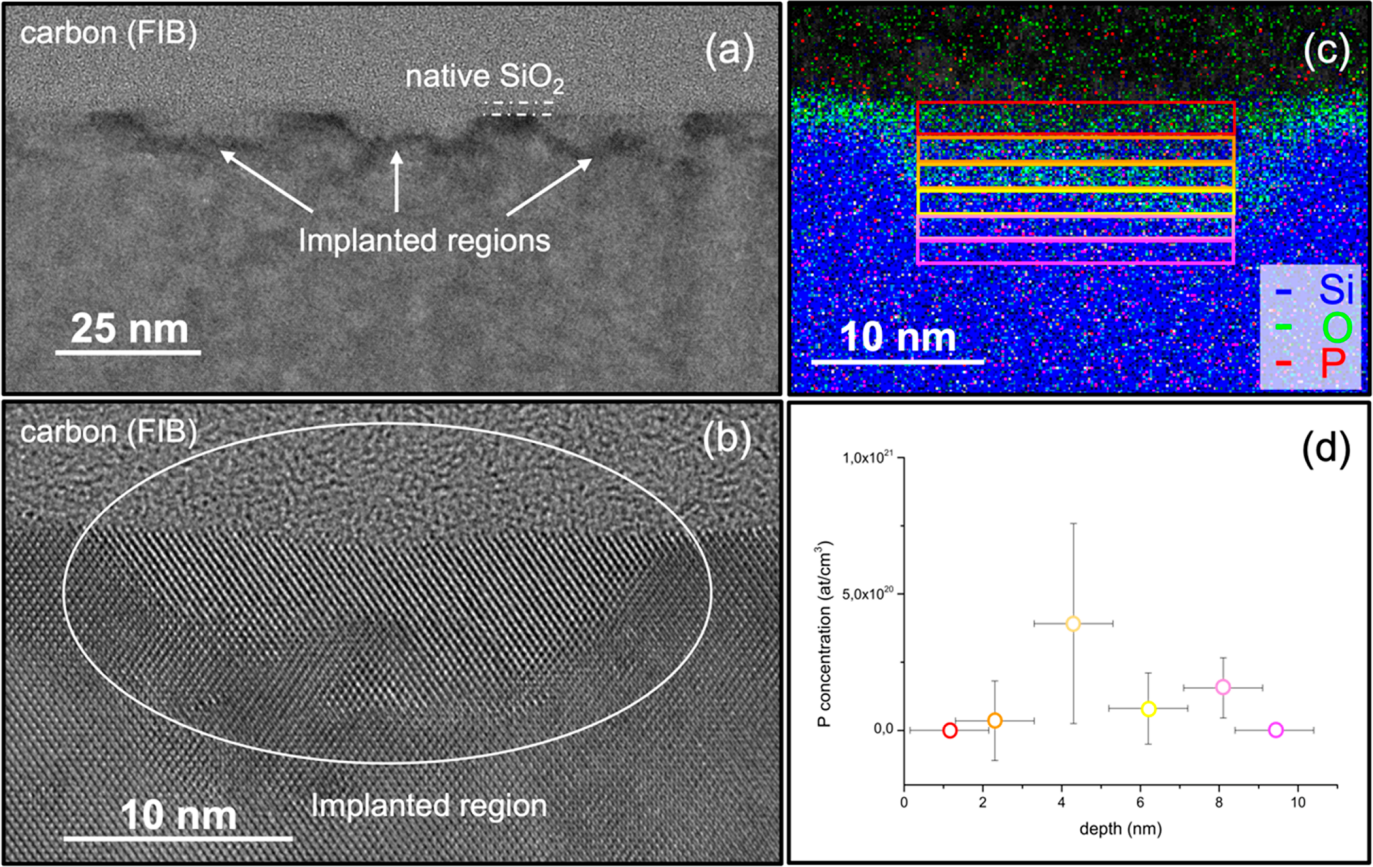
(a) High resolution electron microscopy image of the implanted regions. (b) Zoom at high magnification of one of the implanted regions. (c) STEM-EDX elemental mapping in one of the implanted regions. (d) Average P concentration measured in the rectangles of panel c. Data are reported as a function of depth.

A representative HREM cross-sectional image of the implanted regions is shown in Figure 6a. The implanted regions are conical in shape with lateral dimension of 22–25 nm and center-to-center distance of 36 nm. These values closely match the diameter and periodicity of the pores in the original mesoporous PS template. A 2 nm thick SiO_2_ layer is present at the Si surface. A zoom at high magnification of one of the implanted regions is reported in Figure 6b. The high magnification image confirms that the implanted Si is fully recrystallized, with a perfectly monocrystalline region on the first 4 nm and deeper, a 4 nm thick damaged region, with some extended defects, which likely formed beneath the former amorphous/crystalline interface. Figure 6c displays the STEM-EDX elemental mapping of an implanted region. Although perfectly crystalline, some O atoms can be found in the first 6 nm of the implanted region beneath the 2 nm thick native SiO_2_ layer. Phosphorus concentration is too low for the P to be directly visible in the elemental mapping. Moreover, an erroneous interpretation of the P–K intensity levels may be due to the Pt-M line with energy extremely close to the P–K line. However, when integrating over the thin slices that are represented by the colored rectangles of Figure 6c, phosphorus quantification is possible, although it is important to pay attention to the quantification parameters because of the presence of the Pt-Mα line. Figure 6d plots the average P atomic concentration inside each slice as a function of depth, with the error bar representing the standard deviation obtained by measurement performed on different samples. These values perfectly match the P concentrations in the calibrated phosphorus depth profile obtained by TOF-SIMS analysis and reported in Figure 4a.

**Figure 7:**
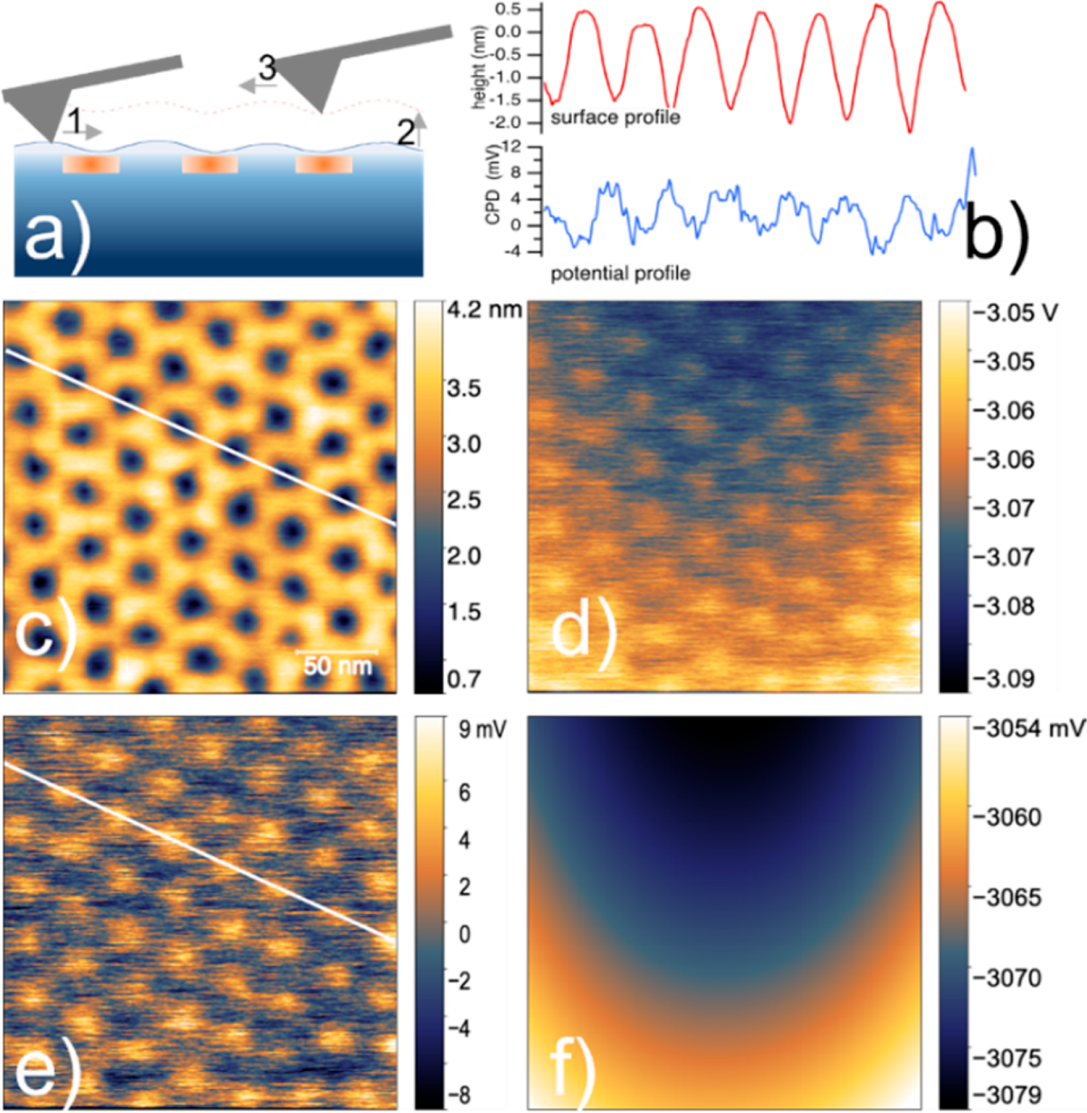
KPFM measurement on sample with 5.07 × 10^14^ cm^–2^ implanted dose after doping activation. (a) Schematic of KPFM measurement: surface morphology is traced first, then the CPD signal is collected while retracing the same scan line at a lift height of 200 nm; (b) signal profile along the surface morphology image (red curve) reported in c) and along local CPD map reported in e (blue curve); (c) surface morphology image; (d) associated as-measured CPD map; (e) local CPD contrast map obtained by subtracting from d the long-range waviness of surface potential around −3 V; (f) the subtracted signal map.

Further information on the distribution and characteristics of implanted regions were obtained by KPFM measurements carried out after the thermal treatment. Figure 7A provides a schematic of the KPFM measurement protocol. Representative measurements of the surface morphology and corresponding potential distribution, taken on the sample implanted with a phosphorus dose of 5.07 × 10^14^ cm^–2^, are reported in Figure 7. The surface morphology at the end of the fabrication process (Figure 7B) has opposite contrast compared to that reported in Figure 3, which was measured after ion implantation and polymer mask removal. Implanted regions are now recessed by ∼1.2 nm with respect to the average surface profile. This feature was observed in all samples after the thermal treatment and confirmed by non-contact AFM measurements using non-conductive sharp silicon probes (Figure S6). The origin of such a change in morphology is not clear. Nevertheless, it is worth noting that in the annealed sample the native oxide was removed before the thermal treatment to promote phosphorus activation. This process step and the successive reoxidation of the silicon surface, which might be inhomogeneous due to the different local doping,^62^ are suggested as the origin of such a change in surface morphology.

The corresponding contact potential map (Figure 7D) shows a long-range waviness plus a short-range regular, rather weak contrast, around the average value of −3 V. To better evidence the short-range features, the long-range component was subtracted (Figure 7F) and the resulting map of the relative CPD contrast is reported in Figure 7E. Considering the spatial arrangement of the brighter spots of Figure 7E, it is obvious to associate them with phosphorus implanted regions; moreover, comparison of the line profiles reported in Figure 7B evidence that these regions at higher potential match the hollows in the morphology profile. On the other hand, the contrast between phosphorus implanted regions and the surrounding p-type silicon is quite weak (∼12 mV), while in an ideal p–n junction array one would naively expect a larger potential difference related to the Fermi level change between the n-doped regions and the p-type silicon wafer. However, KPFM measurements on semiconductor surfaces are not straightforward, as the probe-sample system behaves as a bias-dependent metal–insulator-semiconductor capacitor where probe-sample interaction and local charge state at the semiconductor surface must be taken into account.^63−65^ In fact, as already pointed out by Polak et al.,^63^ the band bending at the semiconductor surface, induced by the presence of surface and interface charges, greatly affects the measured potential difference between n- and p-regions, and voltage differences ≤20 mV were often experimentally observed.^63^ In our system, where no surface passivation step was applied and a defective native oxide likely developed after the thermal treatment, this effect might be further enhanced by the small geometries involved (both in-plane and vertical) and by the high doping level expected in the implanted regions. Incidentally, assuming a work function for the PtSi probe of ∼5 eV, a rough calculation (see the Supporting Information) reveals that a density of ∼10^13^ q/cm^–2^ positive fixed charges at the Si/native oxide interface would account for the measured overall CPD signal of approximately −3 V and, concurrently, for the ∼12 mV difference between p- and n-doped regions. Therefore, although partial activation and/or partial diffusion of implanted phosphorus cannot be definitively excluded, our KPFM measurements are consistent with the successful activation of phosphorus doping in well localized and ordered regions, in a system where a large density of positive surface charges dominates the measured CPD.

**Figure 8:**
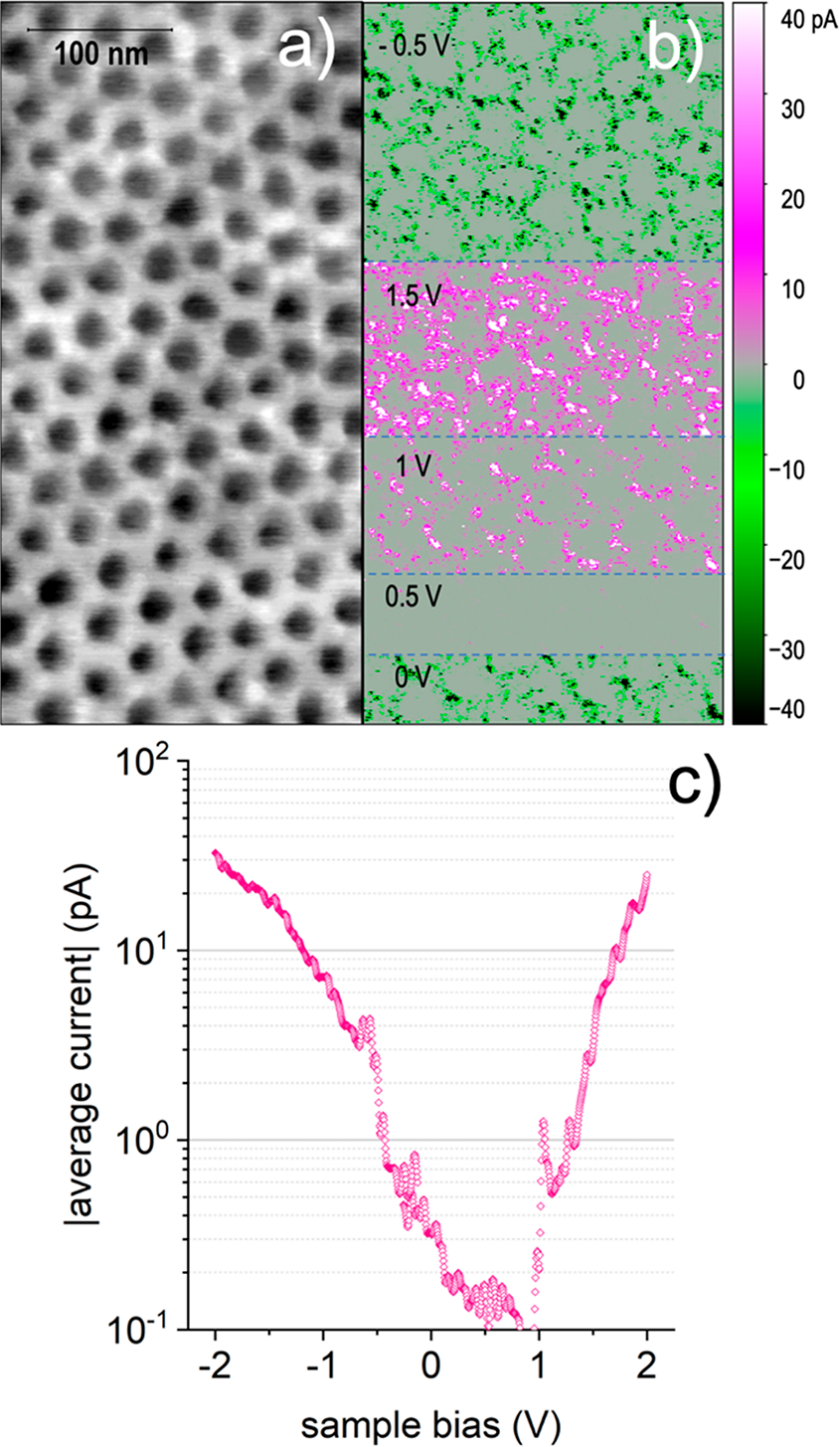
(a) Surface morphology and (b) corresponding current map measured on the sample with a 3.20 × 10^14^ cm^–2^ dose, while changing the sample voltage bias as reported in the image. The grayscale of the morphology image corresponds to 3 nm. (c) Absolute average current vs sample bias voltage in semilog scale; the current data are obtained averaging over 60 *I*–*V* measurements taken at different surface sites.

In the same experimental setup, the local electrical conductivity was investigated by conductive-AFM. Differently from the KPFM setup, C-AFM measurements are taken in contact mode with the probe acting as local top electrode at virtual ground, while the voltage bias is applied to the sample backside; the current flowing between the probe and the sample is collected through an amplifier, concurrently to the sample surface. Figure 8a and 8**b** report a representative C-AFM measurement carried out on the sample implanted with 3.20 × 10^14^ cm^–2^ dose. The surface morphology is similar to that already obtained in KPFM measurements. More interestingly, the corresponding current map shows that, at both positive and negative bias, current flows almost solely through the nonimplanted regions. Comparing the segments measured at 1 and 1.5 V, it is also observed that the current-carrying regions expand with increasing bias. On the other hand, the current collected when the probe passes over the implanted regions is below the detection limit at all applied bias. We also observe that no current is collected at *V*_s_ = 0.5 V, while some current flows at *V* = 0 V. Actually, this result is consistent with the assumption that positive charges are present at the semiconductor surface: a positive bias is required at the sample backside to balance the surface negative band bending induced by these positive charges before current starts flowing. Similar trends were observed on the other samples, also using different probes and measurement conditions.

To further explore the characteristics of the collected current, several current–voltage measurements were acquired at various surface sites. However, we realized that it is not possible to discriminate between measurements taken on implanted and non-implanted regions; in fact, the small size of surface features, which is comparable to the probe contact area, combined with the finite precision of tip positioning, does not allow to clearly distinguish the origin of the collected current. Therefore, we chose to automatically acquire many *I*–*V* curves at regularly spaced surface sites and then to average the results, thus obtaining a statistical trend of the current–voltage characteristics in the ±2 V range, as reported in Figure 8c. The obtained *I*–*V* curve is not symmetric: as already noted for the current map, current minimum lays at positive sample bias voltage, in the interval 0.5–1 V; moreover, above 1 V the current increases fast, following a nearly exponential trend, whereas below 0 V the increase is less steep. The overall impression is that the sample does not behave as a simple resistor and it is influenced to some extent by the presence of the implanted regions. Unfortunately, the C-AFM analysis does not allow further deductions, due to the limited current range and poor measurement reproducibility related to the modification occurring at the probe-sample system during the flow of high-density current.

**Figure 9:**
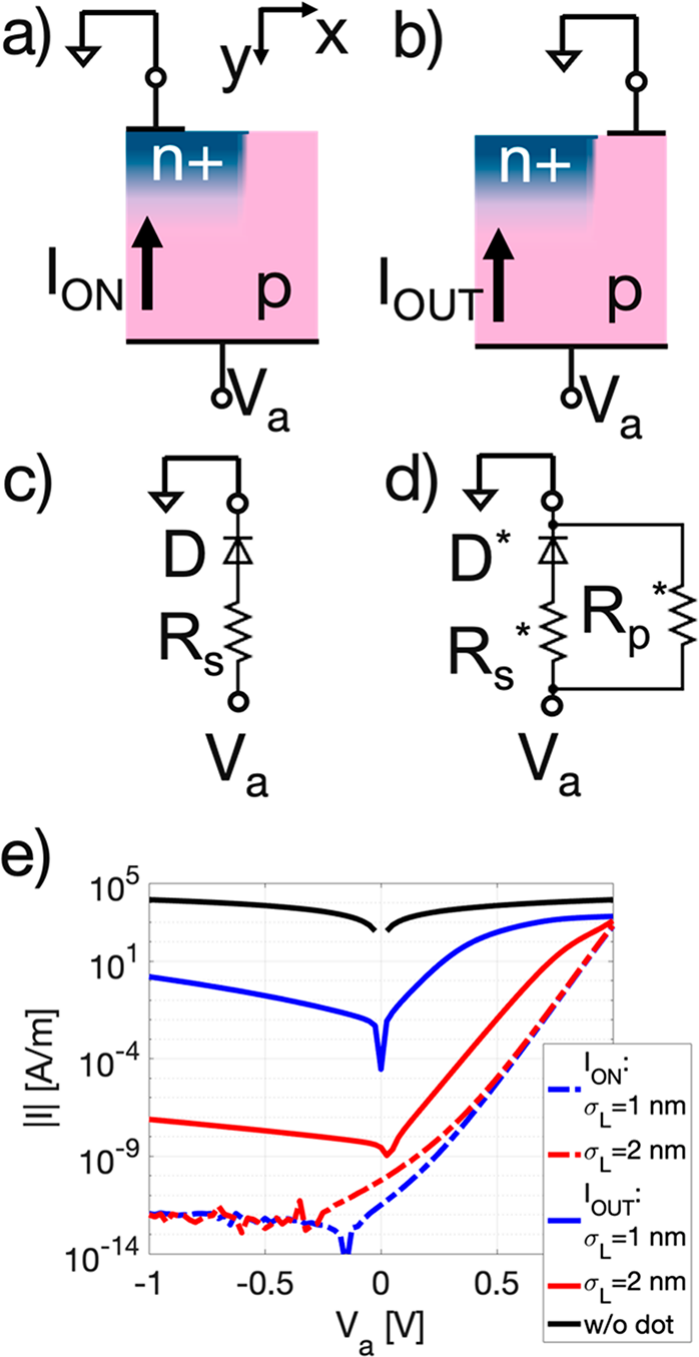
Modeling and simulation results. Two cases were schematized: (a) top contact on the dot and (b) top contact located on the region outside the dot. In the center panels the equivalent circuits are highlighted for (c) the on-dot case and (d) the outside-dot case. (e) The simulated |*I*|−*V* curves are reported for both cases and for two significant values of the σ_L_ = 1 and 2 nm. The curve labeled “w/o dot” shows the *I*_OUT_ if there was no dot so when a simple plain p-region slab is simulated. Note that current is not expressed in A but in A/m due to the 2D dimensionality of the model that does not consider the third dimension (*z* dimension, normal to the plane of the figure).

These experimental results can be interpreted by means of numerical simulations stating that the measured *I*–*V* characteristics are compatible with the electrical behavior of a working p–n nanojunction. To support this statement by simulation results, the sample portion between the n+ well center vertical axis and half the distance between the adjacent n+ well center was modeled as a 2D rectangular domain with lateral size *x*_D_ = 17.5 nm and vertical one *y*_D_ = 30 nm. The domain material was set to be crystalline silicon with a uniform acceptor doping of *N*_A_= 8.5 × 10^18^ cm^–3^ with a heavily n+ doped Si well incorporated in its top-left region. The n+ well featured a Gaussian doping profile with a maximum of donor doping *N*_D_ = 3 × 10^20^ cm^–3^ located at the domain top border with a vertical standard deviation σ_*y*_ = 4.2 nm, as extrapolated from the ToF-SIMS analysis. The radius of the n+ well was set to *r* = 10 nm, leading to a n+ well of nanoscopic size, namely a quantum dot. No information on the lateral drop-off doping profile standard deviation of the dot σ_L_ could be extracted from the experimental data. Ideal ohmic contacts were applied to the top and to the bottom of the domain to set the voltage at these boundaries of the system whereas boundary conditions with zero lateral electric field were applied to the lateral interfaces due to symmetry considerations. A small top contact, with a lateral size *r*_c_ = 5 nm, modeled the radius of the AFM tip. The top contact was set to a ground potential in all the simulations whereas a DC voltage *V*_a_ was applied to the back-contact, as specifically done in the experiments. To reduce the number of AFM tip positions to be modeled, only two main spatial positions of the top contact were considered: in the former the top contact was positioned on top left region of the dot (see Figure 9a) and collects an *I*_ON_ current, whereas in the latter the top contact was set at the top right region (see Figure 9b) collecting an *I*_OUT_ current.

Figure 8 showed a current flow only when the AFM tip was positioned outside the dot whereas almost no current was recorded when it was on the dot. This lack of current can be motivated by guessing that the current levels were below our measurement background noise level. Actually, simulations reported in Figure 9e confirm that *I*_ON_ is very low for negative and small positive *V*_a_, whereas *I*_OUT_ is always significantly higher, thus corroborating the experimental findings. Moreover, the AFM probe whose size is expected to be just smaller than the implanted regions, may collect current from the surrounding nonimplanted regions, especially while measuring local *I*–*V* characteristics; therefore, due to the very small size of the n+ implanted regions and large *I*_OUT_, pure *I*_ON_ cannot be actually accessed in our C-AFM setup. As already pointed out, the *I*–*V* characteristics shown in Figure 8c presents a clear asymmetry. This asymmetry in the shape of the *I*–*V* experimental data is clearly visible also in the simulation results (Figure 9e), whereas the voltage shift of measured *I*–*V* is not accounted, because the presence of the thin top oxide, along with its possible fixed charges, was not included in the model. The asymmetry in the shape can be explained qualitatively by means of simple circuit models. When top contact is on the dot, the *I*–*V* curves can be mapped to those of an equivalent circuit model of a diode in series with its parasitic resistance as sketched in Figure 9c. When top contact is located outside the dot, the diode is poorly biased and charge transport effects in the p-region on the right side of the dot can be modeled as an additional resistor in parallel to the diode (Figure 9d). For *V* < 0, the diode is poorly reversely biased, so the current is shunted mostly through the resistor in parallel with an equivalent resistance that depends strongly on σ_L_. In Figure 9e, we also added the limit case when no dot is formed (see “w/o dot” curve), showing the foreseen linear *I*–*V* behavior in a uniform p-Si slab. When *V* > 0 the diode is forward biased so there is a sum of its direct current and of the current flowing through the equivalent p-region resistor. Here, the current behavior depends on which one of the two components dominates. The balance between the two depends on σ_L_: for σ_L_ = 1 nm, i.e., for a narrow dot, the diode biasing worsens in such a way that the current passing through the resistor in parallel has a higher impact on the final *I*–*V* curve. For a larger σ_L_ = 2 nm, i.e., for a slightly larger dot, there is a better biasing of the junction, so the *I*–*V* characteristic is closer to the common one of a diode. Therefore, the experimental results can be qualitatively mapped on the simulation ones of a not ideal p–n nanojunction featuring a very small σ_L_.

## Conclusions

In conclusion, this work demonstrated that mesoporous thin films, obtained by BCP self-assembly, efficiently shield a substrate during ultralow energy (3 keV) implantation of phosphorus ions at high doses. The mesoporous PS soft mask exhibited no modification of the morphology and no detectable damage after the implantation process. The phosphorus atoms trapped in the mask linearly increase as the implantation dose increases, confirming the capability of the PS matrix to properly retain the low energy phosphorus ions in the dose range under investigation. The structural and compositional characterization of the samples upon removal of the mesoporous PS template demonstrated that the phosphorus ions were implanted into the silicon substrate throughout the mask, leading to localized implantation in correspondence of the pores of the PS film. Raman spectra suggested the presence of a thin layer of amorphous Si in the implanted samples. A low temperature thermal treatment at 650 °C was demonstrated to effectively promote silicon recrystallization and dopant activation without detrimental effects on their spatial confinement. These observations were further corroborated by specific SPM measurements. In particular, KPFM measurements showed an ordered modulation of the surface potential signal, compatible with the formation of localized n-doped regions. C-AFM measurements indicated that these regions hinder the current flow, at least in the explored voltage range. Conversely asymmetric *I*–*V* curves were obtained outside the implanted regions. According to FEM simulations, measured *I*–*V* characteristics are fully compatible with a not ideal but working p–n nanojunction with a lateral drop-off doping profile in the very few nanometers range.

The collected results demonstrate the possibility to locally modify the potential landscape and conductivity of the semiconductor substrate by the introduction of a periodic array of dopants. The localization of dopants in very small nanovolumes paves the way to several applications like, for instance, the engineering of the semiconductor band structure, the synthesis of artificial crystals, or the formation of quantum dot arrays in a semiconductor host matrix.

## Supporting Information Available

Diameter of the pores in PS as a function of implantation dose; phosphorus depth profiling in the polymeric mask; AFM measurement of surface roughness before implantation; phosphorus depth profiling and Raman spectra of the substrate as a function of annealing temperature; AFM morphology of the substrate after implantation; estimation of the contact potential difference (PDF)
