# Supplementary material for: Periodic Arrays of Dopants in Silicon by Ultralow Energy Implantation of Phosphorus Ions through a Block Copolymer Thin Film
Source: ACS Appl Mater Interfaces. 2023 Jun 14;15(50):57928–40. doi: 10.1021/acsami.3c03782 (PMC10739587; doi:10.1021/acsami.3c03782)
Supplement: Supplementary file 1 — am3c03782_si_001.pdf [file am3c03782_si_001.pdf]

Periodic arrays of dopants in silicon by ultra-low energy implantation of phosphorus ions through a block copolymer thin film

Stefano Kuschlan,[(1)(2)] Riccardo Chiarcos,[(2)] Michele Laus,[(2)] Francesc Pérez-Murano,[(3)] Jordi Llobet,[(3)] Marta Fernandez-Regulez,[(3)] Caroline Bonafos,[(4)] Michele Perego,[(1),*] Gabriele Seguini,[(1)] Marco De Michielis,[(1)] Graziella Tallarida.[(1)]

(1) *CNR-IMM, Unit of Agrate Brianza, Via C. Olivetti 2, I-20864 Agrate Brianza, Italy.*

(2) *Università del Piemonte Orientale "A. Avogadro", Viale T. Michel 11, I-15121 Alessandria, Italy*

(3) Institute of Microelectronics of Barcelona (IMB-CNM, CSIC), Bellaterra 08193, Spain

(4) CEMES-CNRS, Université de Toulouse, CNRS, 31055 Toulouse, France

Correspondence to: michele.perego@mdm.imm.cnr.it

Supporting Information

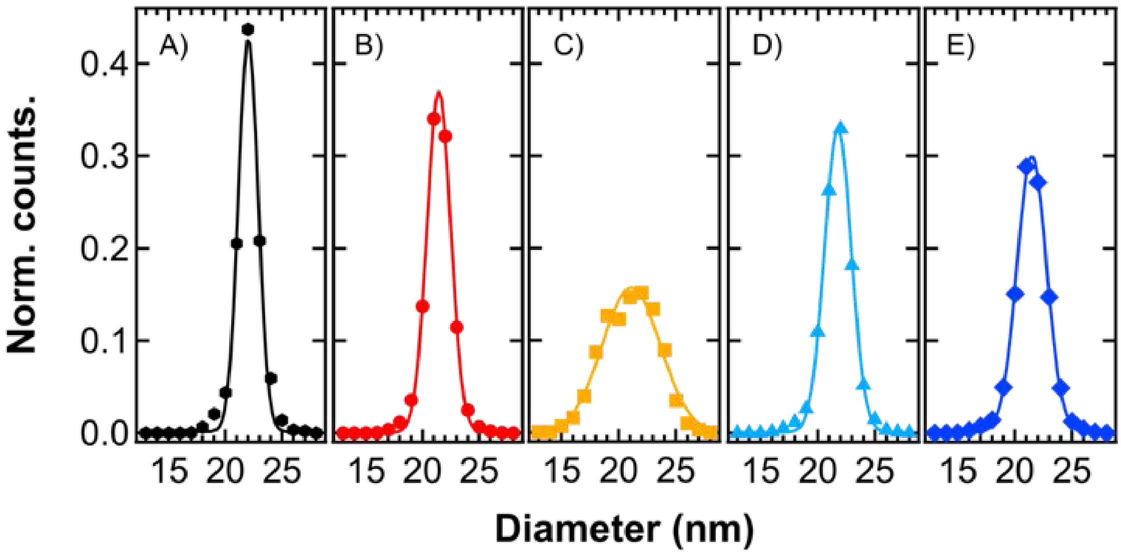

Figure S1. *Distribution of diameters, solid lines are the gaussian fit of the experimental data. A) Pristine PS mask, B) 1.55 x 10^{14} cm^{-2} dose, C) 2.83 x 10^{14} cm^{-2} dose, D) 3.20 x 10^{14} cm^{-2} dose, E) 5.07 x 10^{14} cm^{-2} dose.*

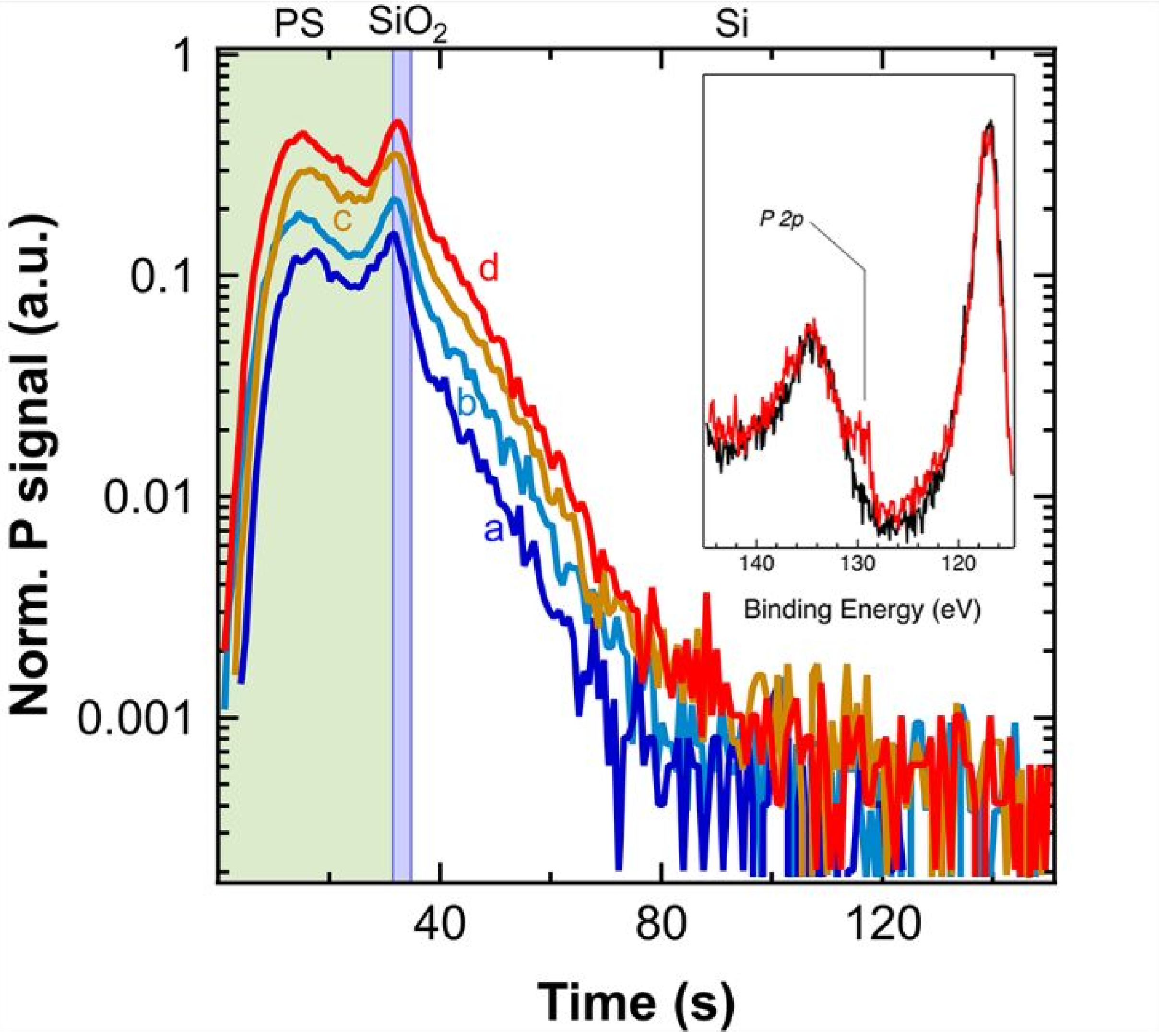

Figure S2. *Depth profile of P after implantation and before removal of PS mask. a) 1.55 x 10^{14} cm^{-2} dose, b) 2.83 x 10^{14} cm^{-2} dose, c) 3.20 x 10^{14} cm^{-2} dose, d) 5.07 x 10^{14} cm^{-2} dose. In the inset, high resolution XPS spectrum of the pristine Si substrate and of the sample implanted with P ions at 3 keV with fluence 5.07 x 10^{14} cm^{-2}.*

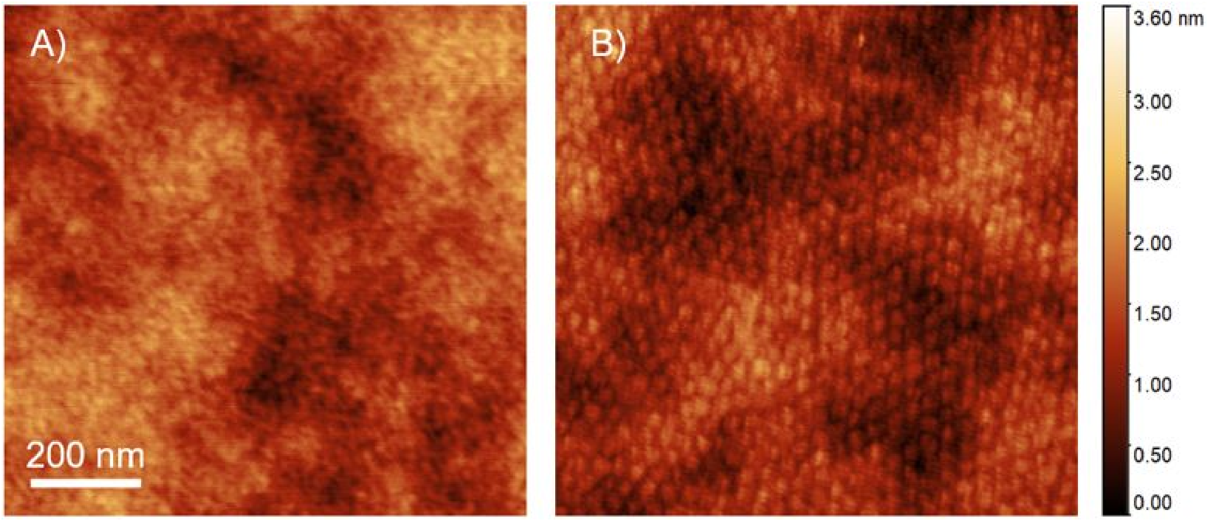

Figure S3. *AFM image of the silicon surface of (A) a pristine substrate, (B) a sample implanted through BCP mask with a dose of 1.55 x10^{14} cm^{-2}.*

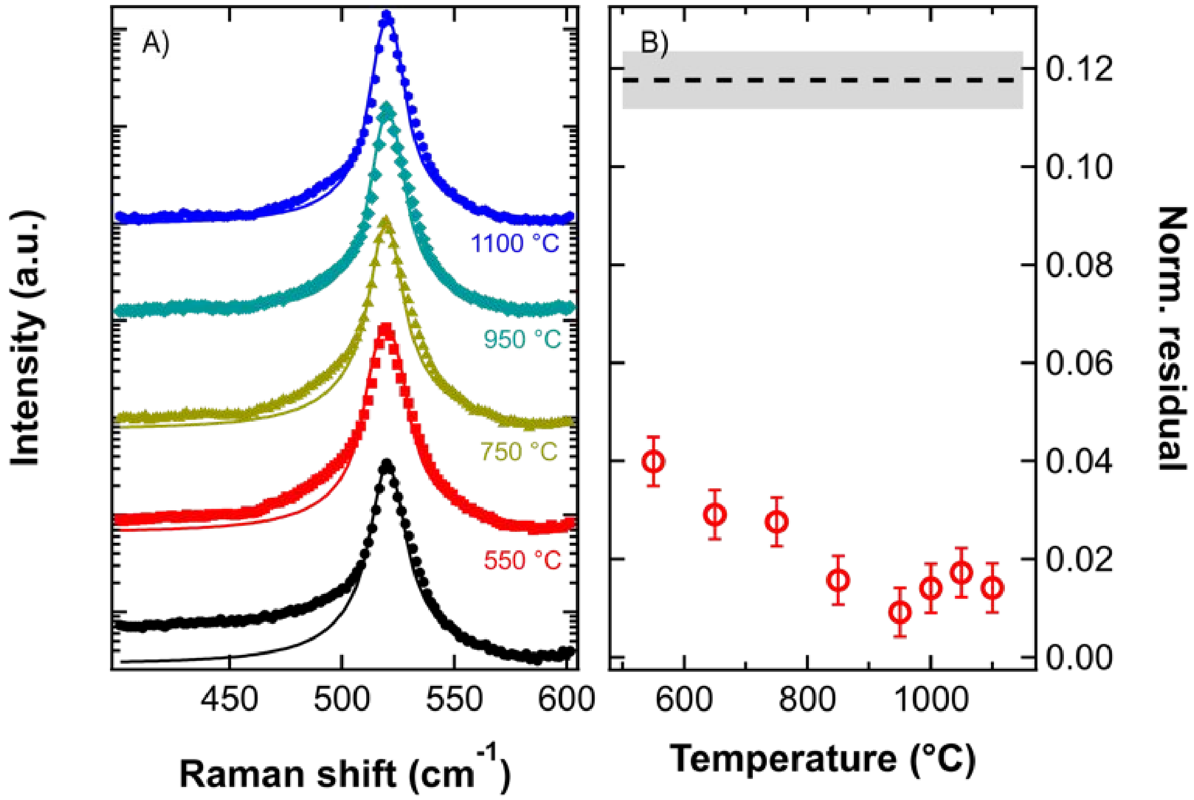

Figure S4. *A) Raman spectra of the sample implanted with dose of 1.55 x 10^{14} cm^{-2}. In the graph spectra are reported before the annealing and after a 5 s annealing at different temperatures. Solid lines represent the fit with a Voigt function. B) Normalized residuals of the [400,500] cm^{-1} interval as a function of the annealing temperature. The black dashed line reports the value with error (grey area) of the sample before the annealing.*

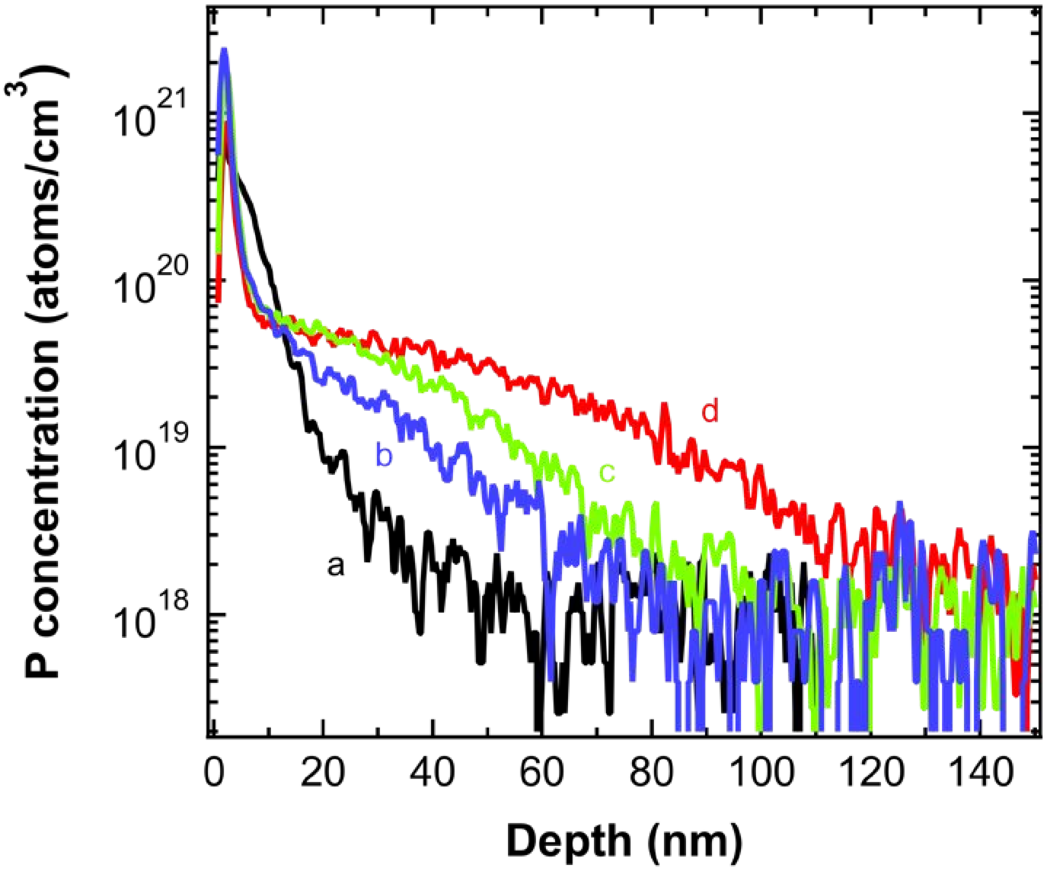

Figure S5. *Calibrated profile of P for the sample implanted with dose 1.55 x 10^{14} cm^{-2}. Curves represent the sample (a) before the annealing, after 5 s annealing at (b) 1000 °C, (c) 1050 °C, (d) 1100 °C.*

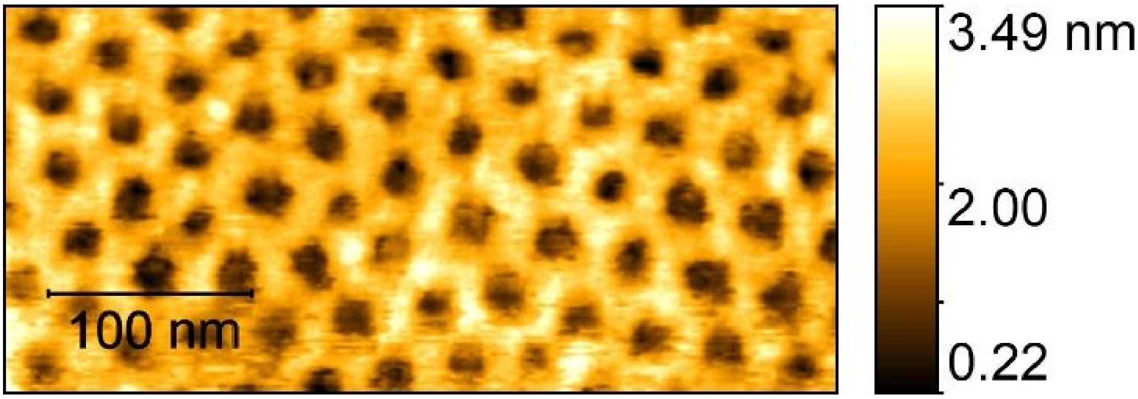

Figure S6. *Surface morphology after the annealing step of the sample implanted with dose 3.2 10^{14} cm^{-2}; the presence of hollows ~1.2 nm deep in correspondence of the implanted regions is confirmed by AFM measurements taken in tapping mode, using uncoated sharp silicon probes (nominal tip radius ~7nm)*

Estimation of the Contact Potential Difference

The surface potential of a semiconductor is influenced by several factors: bulk doping, charge stored at the surface and/or the native oxide, interface defect states, formation of a space charge region, surface band bending.

A general expression for the potential difference between the KPFM probe and the semiconductor surface is [1s]:

$$\Delta V = (V_m - \Phi_m) - V_i - (\phi_s - \Phi_s) \qquad \text{Eq. 1}$$

Where V_m is the voltage applied to the probe;

$\Phi_{m/s}$ are the local workfunction for the metal and the semiconductor, respectively.

V_i is the potential drop across the insulator (the native oxide, in our system) generated by the surface charges;

ϕ_s is the surface potential due to the space charge region;

The KPFM feedback loop adjusts V_m to achieve $\Delta V=0$, thus providing the contact potential difference as:

$$CPD = -V_m = -\left(\frac{\Phi_{ms}}{q} + \phi_s + V_i\right) \qquad \text{Eq. 2}$$

$$\Phi_s = \chi + \frac{E_g}{2} - \pm\, \phi_F \qquad \text{Eq. 2b}$$

$$\phi_F = \pm\, KT \ln \frac{N_{dop}}{n_i} \qquad \text{Eq. 2c}$$

(Eq. 2c is positive for n-type and negative for p-type)

While scanning between n- and p-doped regions, assuming that V_i , Φ_m and the silicon electron affinity, χ, are constant across the surface, the CPD difference can be written as:

$$\Delta CPD^{n,p} = \Delta V_k$$

Where ΔV_k depends on the doping level and accounts for the band bending at the surface induced by the presence of surface and interface charges. In other words, in relation to eq 2, ΔV_k includes the variable part of Φ_s due the Fermi level adjustment at the surface, and ϕ_s due to the presence of surface/interface charges.

ΔVk was estimated by Polak et al [2s] in a wide range of doping levels and surface/interface charge densities. Considering the doping densities relevant for our samples (Nd=5x10^{20} cm^{-3} and Na=8.5x10^{18} cm^{-3}), a ΔVk~12meV (as detected in our measurements) is obtained with a surface charge density of ~10^{13}cm^{-2}

Going back to Eq 2 and assuming:

Φ_m/q = 5 eV [3s]

χ/q = 4.05 eV

$V_i = \frac{x_0 Q_f}{K_i \epsilon_0}$ where Q_f is an effective fixed charge density centered at a plane distance x_0=~1 nm from the interface, K_i=3.9 is the dielectric constant of the native oxide and ε_0 is the vacuum permittivity [1s];

We find that, assuming Q_f =5x10^{13} cm^{-2}, CPD~ -3V, which is the experimental background value detected by KPFM (Fig. 8f).

Although this computation is quite rough and not fully self-consistent, KPFM results indicate that our samples can be thought as arrays of p-n nanojuctions with a surface native oxide characterized by a high density of positive fixed charges that strongly affect the semiconductor surface band bending.

1s. Bonilla, R. S. Modelling of Kelvin Probe Surface Voltage and Photovoltage in Dielectric-Semiconductor Interfaces. Mater. Res. Express 2022, 9 (8). https://doi.org/10.1088/2053-1591/ac84c8.

2s. Polak, L.; Wijngaarden, R. J. Two Competing Interpretations of Kelvin Probe Force Microscopy on Semiconductors Put to Test. Phys. Rev. B 2016, 93 (19), 1–10. https://doi.org/10.1103/PhysRevB.93.195320.

3s. Niranjan, M. K.; Zollner, S.; Kleinman, L.; Demkov, A. A. Theoretical Investigation of PtSi Surface Energies and Work Functions. Phys. Rev. B - Condens. Matter Mater. Phys. 2006, 73 (19), 1–7. https://doi.org/10.1103/PhysRevB.73.195332.
